# Complement Component C3 Binds to the A3 Domain of von Willebrand Factor

**DOI:** 10.1055/s-0038-1672189

**Published:** 2018-09-26

**Authors:** Jennifer G. Nolasco, Leticia H. Nolasco, Qi Da, Sonya Cirlos, Zaverio M. Ruggeri, Joel L. Moake, Miguel A. Cruz

**Affiliations:** 1 Section of Cardiovascular Research, Department of Medicine, Baylor College of Medicine, Houston, Texas, United States; 2 Department of Bioengineering, Rice University, Houston, Texas, United States; 3 Center for Translational Research on Inflammatory Diseases (CTRID), Michael E. DeBakey VA Medical Center, Houston, Texas, United States; 4 Department of Molecular Medicine, MERU-Roon Research Center on Vascular Biology, The Scripps Research Institute, La Jolla, California, United States

**Keywords:** complement component C3, von Willebrand factor, alternative complement pathway, thrombosis, inflammation

## Abstract

von Willebrand factor (VWF) is a multimeric protein composed of monomeric subunits (∼280 kD) linked by disulfide bonds. During hemostasis and thrombosis, ultralarge (UL) VWF (ULVWF) multimers initiate platelet adhesion. In vitro, human C3 binds to ULVWF multimeric strings secreted by and anchored to human endothelial cell to promote the assembly and activation of C3 convertase (C3bBb) and C5 convertase (C3bBbC3b) of the alternative complement pathway (AP). The purified and soluble C3 avidly binds to recombinant human VWF A1A2A3, as well as the recombinant isolated human VWF A3 domain. Notably, the binding of soluble human ULVWF multimers to purified human C3 was blocked by addition of a monovalent Fab fragment antibody to the VWF A3 domain. We conclude that the A3 domain in VWF/ULVWF contains a docking site for C3. In contrast, purified human C4, an essential component of the classical and lectin complement pathways, binds to soluble, isolated A1, but not to ULVWF strings secreted by and anchored to endothelial cells. Our findings should facilitate the design of new therapeutic agents to suppress the initiation of the AP on ULVWF multimeric strings during thrombotic and inflammatory disorders.

## Introduction

von Willebrand factor (VWF) is a multimeric protein composed of monomeric subunits (∼280 kD) linked into large polymers by disulfide bonds. VWF multimers are synthesized in endothelial cells (ECs) and megakaryocytes, where the multimers are stored in Weibel–Palade bodies (ECs) or α granules (megakaryocytes and platelets). 1 During hemostasis and thrombosis, stimulated human vascular ECs secrete and anchor ultralarge (UL) VWF (ULVWF) multimers in hyperadhesive long string-like structures that initiate platelet adhesion. 2 3

Recently, we showed that EC-secreted/anchored ULVWF string-like structures, in addition to initiating platelet adhesion, serve also as surfaces for assembling the components of the alternative complement pathway (AP). 4 In vitro, both hydrated C3 [C3(H _2_ O)] (designated in this paper as “C3”) and C3b (C3 after cleavage of the small peptide, C3a) are active forms of C3 that share domains enabling them to attach to surfaces and initiate activation of the AP. 5 6 Both hydrated C3 (C3) and C3b bind to EC-anchored ULVWF multimeric strings and initiate assembly on the strings of C3 convertase (C3bBb) and C5 convertase (C3bBbC3b). 4 This may be an important molecular mechanism for initiation and activation of the AP. In contrast, there is little or no binding of C4 to EC-anchored ULVWF strings, 4 indicating that the classical and lectin complement pathways, which require C4, are not also activated under these conditions.

To identify the precise location for C3 binding to VWF, we investigated the interaction of C3, C3b, and C4 with VWF A1, A2, and A3 domains either linked together as VWF A1A2A3 or as individual, isolated recombinant A1, A2, or A3 domains. The recombinant VWF A1A2A3 protein is referred to in figures as “TD.” We identified VWF A3 domain as containing the docking site for C3. The results reported here will help clarify the molecular mechanisms underlying the cross-talk between coagulation and complement activation relevant in host defense. This will help design new therapeutic agents to suppress activation of the AP on ULVWF strings during thrombotic and inflammatory disorders.

## Materials and Methods

### Antibodies and Proteins

We used monoclonal anti-His antibody (GenScript, New Jersey, United States), goat anti-VWF antibody (A80–138, Bethyl Labs, Texas, United States), rabbit anti-VWF antibody (Ramco Laboratories, Texas, United States), horseradish peroxidase (HRP) conjugated goat anti-rabbit immunoglobulin G (IgG) and rabbit anti-goat IgG (Pierce Antibodies, Illinois, United States), and a CM series sensor chip (GE Health Care, New Jersey, United States), Antibody MR-5 is a mouse monoclonal IgG1 that binds to a specific sequence (residues 1711–1761) in the A3 domain of human VWF. 7 It was produced at The Scripps Research Institute, where heavy and light chain of the IgG were cloned from hybridoma cDNA and engineered for expression as monovalent Fab in *Drosophila melanogaster* S2 cells. Highly purified C3, C3b, and C4, as well as monospecific anti-C3 and anti-C4 antibodies, were purchased from Complement Technologies (TX). Recombinant VWF A1A2A3 with His tag was expressed (and glycosylated) in mammalian cells (HEK293T) and purified as previously described. 8 Recombinant individual VWF A1, A2, and A3 domains with His tag were expressed in *Escherichia coli* (in nonglycosylated forms) and purified as described. 9 VWF multimers enriched in ULVWF forms were obtained as we previously described 10 11 from histamine-stimulated cultured human umbilical vein EC supernatant, and concentration was determined using a VWF enzyme-linked immunosorbent assay (ELISA) kit (Aviva System Biology, California, United States).

### ELISA Binding Assay

Recombinant human VWF A1A2A3 or recombinant human individual A1, A2, or A3 domains were incubated overnight with purified human C3, C3b, or C4 at 4°C to allow complex formation. The complexes were captured with anti-His antibody and detected by anti-C3 or anti-C4, HRP-conjugated secondary anti-IgG antibody, and the chromogenic substrate, 3,3′,5,5′-tetramethylbenzidine (TMB, Life technologies, Massachusetts, United States) was used for detection and quantification. The color reaction was read at 450 nM in a spectrophotometer.

Specifically, 100 μL of the mixtures of VWF domains and C3, C3b, or C4 were pipetted in duplicate and then incubated in High Bind Stripwells (Greiner Bio-One, Germany) with immobilized mouse monoclonal anti-His tag antibody (160 ng/mL) (Genscript, NJ) for 2 hours at 37°C. Either VWF A1A2A3 or any of the individual A domains with bound C3, C3b, or C4 was captured by the anti-His antibody immobilized onto the wells. Bound C3, C3b, or C4 (10 μg/mL) to 100 μg/mL of either the VWF A1A2A3 or individual A1, A2, or A3 domain was detected by either goat anti-human C3 or anti-C4 plus rabbit anti-goat antibody linked to HRP (Pierce Antibody, Illinois, United States). Detection and quantification in the spectrophotometer was with TMB, as above. Bovine serum albumin in phosphate-buffered saline (PBS; 1%) was used as negative control.

### Binding Kinetics of Complement Proteins and Recombinant VWF Domains

Surface plasmon resonance (SPR) binding studies were performed using a BIAcore 3000 system (BIAcore, Piscataway, New Jersey, United States), as previously described. 12 13 Either 50 μg/mL of C3, C4, or a VWF A domain protein in 50-mM sodium acetate (pH 5.0) was covalently coupled via amine coupling to a sensor chip (CM5) as directed by the manufacturer. The binding assays were performed in 10-mM HEPES, 150-mM NaCl, 0.005% Tween-20, pH 7.4 at 25°C at a flow rate of 10 µL/min. An activated blank channel was used as control for nonspecific binding correction. Binding at equilibrium was determined at a series of concentrations of the perfused protein (C3, C4, or a VWF A domain) at 0.0, 0.05, 0.1, 0.25, 0.5, 0.75, 1.0, 1.5, and 2.0 μM. Kinetic rate constants were determined by using BIA evaluation software (version 3.0) supplied by the manufacturer.

### Bio-layer Interferometry

We used bio-layer interferometry (BLI; Octet Red 384; Pall ForteBio LLC) to demonstrate the interaction of C3 with VWF enriched with ULVWF multimers (VWF/ULVWF or ULVWF). The C3 protein was biotinylated and captured using the High Precision Streptavidin (SAX) Biosensor, following the manufacturer's instruction. Sensors with captured C3 protein were dipped and read onto solutions containing VWF/ULVWF multimers (40 ng/mL) mixed with either mouse control isotype IgG (25 μg/mL) or MR-5 (25 μg/mL) in PBS for 400 seconds.

## Results

**Fig. 1:**
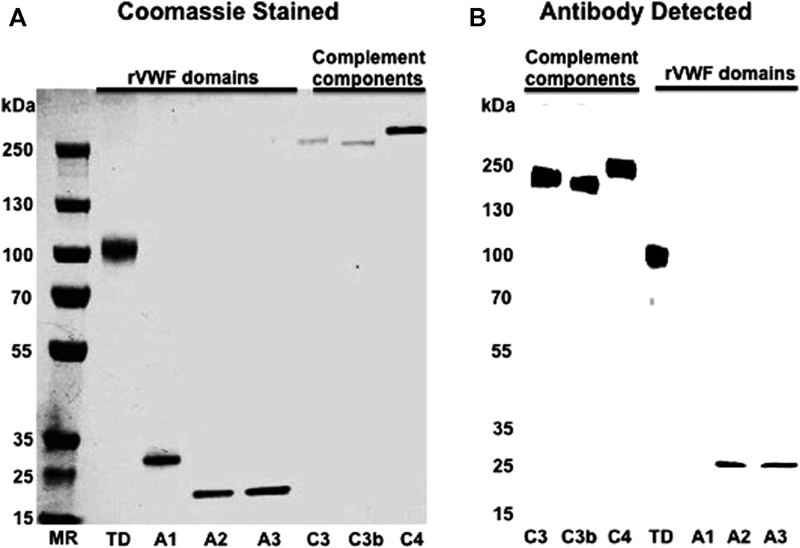
Gel display of VWF and complement proteins. In ( **A** ) recombinant human VWF A1A2A3 (TD), individual VWF A1, A2, and A3 domains, and purified human C3, C3b, and C4 were electrophoresed into a 4–12% SDS-polyacrylamide gradient gel (top left panel) and the protein bands were stained with Coomassie Blue. In ( **B** ), the same samples as in ( **A** ) were transferred onto an Immobilon-P transfer membrane (PVDF), and detected using rabbit anti-VWF plus secondary goat anti-rabbit-HRP; or with goat anti-C3 (for C3 and C3b) or goat anti-C4 plus secondary rabbit anti-goat-HRP. (Note: our rabbit polyclonal anti-VWF antibody does not detect the VWF A1 domain.)

In Fig. 1 , the proteins used in this study are displayed by Coomassie Blue staining and western blotting using monospecific antibodies. The recombinant His-tagged VWF A1A2A3 and recombinant individual His-tagged VWF A1, A2, and A3 domains, as well as C3, C3b, and C4, are all in highly purified form. (The rabbit anti-VWF that we used does not recognize the VWF A1 domain.)

**Fig. 2:**
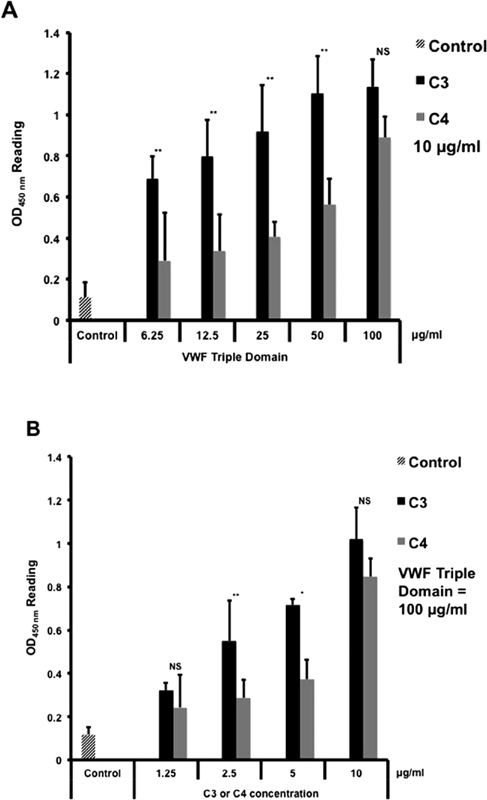
Recombinant VWF-A1A2A3 triple domain binds to purified C3 and, to a lesser extent, purified C4. In preliminary experiments using one immobilized protein and one soluble protein, we determined the concentration ratios that yielded maximum binding. In ( **A** ), a constant concentration of either C3 ( *n* = 5) or C4 ( *n* = 5) was premixed with a range of VWF A1A2A3 triple domain concentrations before incubation in microwells with immobilized mouse monoclonal anti-His-antibody (160 ng/mL) to capture the His-tagged VWF A1A2A3. Subsequently, C3 or C4 bound to the VWF A1A2A3 was detected using goat anti-human C3 or goat anti-human C4 plus rabbit anti-goat HRP-tagged secondary antibody and TMB. Our rabbit anti-human VWF does not recognize the VWF A1 domain (see Fig.1 ); consequently, to capture with maximum effectiveness, His-tagged VWF A1A2A3 (VWF triple domain) with bound C3 or C4, we used mouse monoclonal anti-His antibody. In ( **B** ), the opposite experiments were also done. Anti-C3 or anti-C4 were immobilized on ELISA plates to capture a series of C3 or C4 concentrations preincubated with a constant concentration of 100 μg/mL of recombinant VWF A1A2A3. The C3 or C4 bound VWF A1A2A3 was detected using rabbit anti-human VWF and a secondary goat anti-rabbit/HRP. The color reaction produced by 3,3′,5,5′-tetramethylbenzidine was read in spectrophotometer at A450. A constant concentration of VWF A1A2A3 was premixed with a range of C3 ( *n* = 7) or C4 ( *n* = 7) concentrations before incubation and analysis as in ( **A** ). Controls were PBS/1% bovine serum albumin (BSA), VWF A1A2A3, A1, A2, or A3 alone, or purified C3 or C4 alone at the same concentrations used in the mixes ( *n* = 12). *p* -Value was calculated using Student's *t* -test. * *p* < 0.05; ** *p* < 0.01.

Fig. 2 demonstrates the results of binding studies between VWF domains and complement components, as determined using an ELISA technique with immobilized anti-His antibody. Constant concentrations of either soluble C3 or C4 (10 μg/mL) were bound during prolonged incubation to a range of concentrations of His-tagged VWF A1A2A3 ( Fig. 2A ). At all concentrations tested of the VWF triple domain, binding of C3 exceeded the binding of C4. Similar results were obtained when a constant concentration of soluble His-tagged VWF A1A2A3 (100 μg/mL) was incubated under the same conditions with a range of concentrations of C3 or C4 ( Fig. 2B ).

**Fig. 3:**
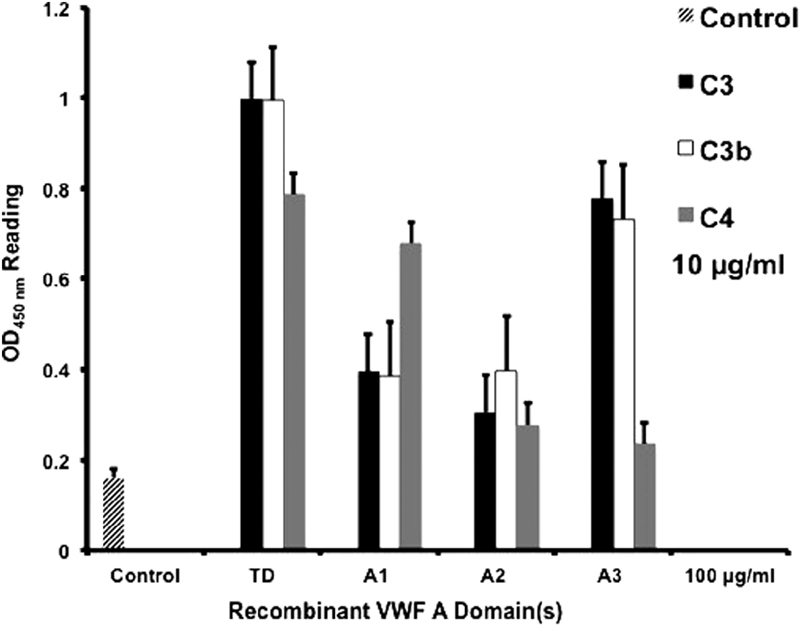
Purified human C3 and C3b bind predominantly to recombinant human VWF A3, and purified human C4 binds predominantly to recombinant VWF A1. 100 μg of each VWF A domain were mixed separately with 10 μg/mL of either C3, C3b, or C4, and incubated at 4°C overnight. The mixtures were pipetted into microwells containing immobilized rabbit anti-His antibody, and incubated for 2 hours at 37°C. The wells were washed and the quantity of C3 or C4 bound to each domain was determined using either goat anti-human C3 or C4 antibodies, as described in Fig. 2 ( *n* = 7). Mixtures of recombinant human VWF A1A2A3 (TD) were used as positive controls. The negative control was phosphate-buffered saline (PBS)/bovine serum albumin, pH 7.4. p-Value was calculated using Student's *t* -test. * *p* < 0.05; ** *p* < 0.01.

Using the individual soluble recombinant VWF A1, A2, and A3 domains, and the same prolonged incubation before ELISA, it was determined that soluble C3 and C3b bind predominantly to soluble VWF A3. In contrast, soluble C4 binds predominantly to soluble VWF A1 ( Fig. 3 ). There was little binding of either C3 or C4 to the VWF A2 domain.

**Fig. 4:**
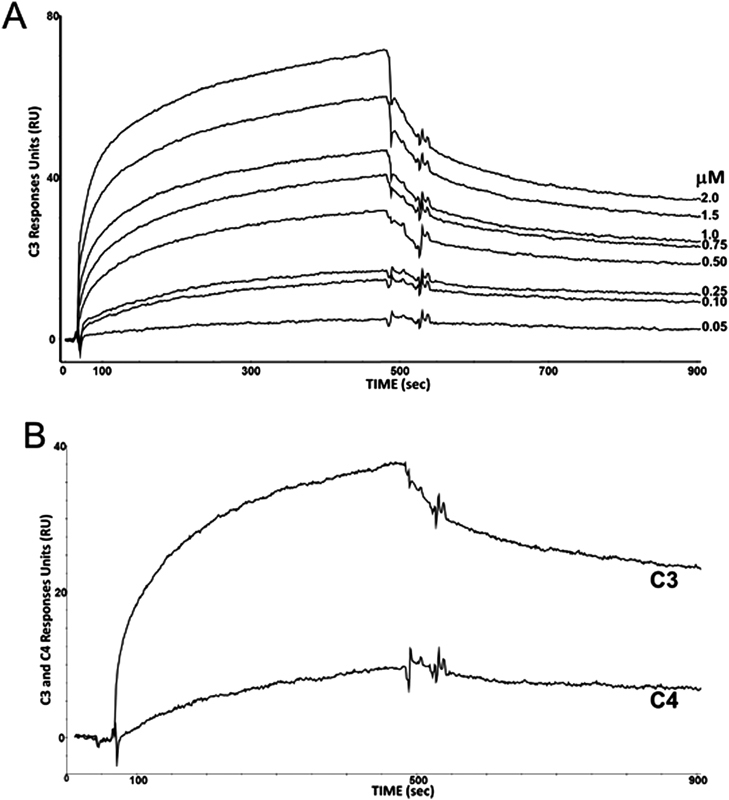
Analysis of the binding of C3 and C4 to immobilized VWF-A3 domain using surface plasmon resonance. ( **A** ) A concentration range ( *as indicated* ) of C3 was analyzed across a recombinant VWF-A3 domain coupled to a biosensor surface at a flow rate of 10 μL/min. C3 bound to VWF A3 domain with a *K*_D_ of 137 nM. ( **B** ) Sensorgrams represent the binding of C3 (500 nM) and C4 (500 nM) to immobilized recombinant VWF-A3 domain. The C4 had a *K*_D_ > 2.0 × 10 ^3^ nM, indicating a binding affinity for VWF A3 domain lower than C3 protein.

Surface plasmon resonance experiments were conducted to determine the binding of purified C3 or C4 to recombinant VWF A1A2A3 or individual A domains ( Table 1 and Fig. 4 ). (Lower *K*_D_ values indicate higher protein–protein affinity.) The data demonstrate that: (1) C3 binds to VWF A1A2A3 with an affinity that is several-fold greater than the affinity of C4; (2) using individual A domains, C3 binds predominantly to A3 and C4 binds predominantly to A1; and (3) neither C3 nor C4 binds to A2. These findings are compatible with our results obtained in the ELISA experiments.

**Table 1:** Surface plasmon resonance binding

Immobilized ligand	Analyte soluble	*K*_D_ (nM)	Number of experiments
VWF A1A2A3	C3	661 ± 290	4
VWF A-1	C3	252 ± 129	5
VWF A-2	C3	No binding	2
VWF A-3	C3	137 ± 88	7
VWF A1A2A3	C4	>2 × 10 ^3^	4
VWF A-1	C4	139 ± 65	8
VWF A-2	C4	No binding	3
VWF A-3	C4	>2 × 10 ^3^	4
C3	VWF A1A2A3	232 ± 46.1	5
C3	VWF A-1	>2 × 10 ^3^	5
	VWF A-2	No binding	2
	VWF A-3	100 ± 26	4
C4	VWF A1A2A3	523 ± 64	8
C4	VWF A-1	504 ± 132	5
	VWF A-2	No binding	2
	VWF A-3	>2 0× 10 ^3^	4

Note: The 1:1 Langmuir binding model and the shift baseline model were used to calculate the dissociation constants ( *K*_D_ ).

Both the 1:1 Langmuir binding model and the shift baseline model were used to fit the kinetic data shown in Table 1 for the binding interaction between C3 and C4 across the separate flow cell surface for VWF A1A2A3 and the individual domains, A1, A2, and A3. Dissociation rates ( *K*_D_ ) and *n* (number of experiments performed) are also shown. Kinetic analysis shows significantly higher affinity of C3 than C4 for binding to VWF A1A2A3 ( Table 1 ). C3 ( *K*_D_ = 661 ± 290 nM) exhibited a much lower *K*_D_ than C4 ( *K*_D_ > 2 × 10 ^3^ nM) ( Table 1 ). Similarly, binding of VWF A1A2A3 to immobilized C3 ( *K*_D_ = 232 ± 46.1 nM) showed higher affinity than binding to immobilized C4 ( *K*_D_ = 523 ± 64 nM) ( Table 1 ).

The individual A3 domain had a binding preference for C3 over C4, as determined by kinetic analyses. From Table 1 , the *K*_D_ for the binding of soluble A3 domain to immobilized C3 was 100 ± 26 nM, and a similar *K*_D_ of 137 ± 88 nM was determined for the binding of soluble C3 to immobilized A3 domain. On the other hand, A3 had a weak binding affinity for C4 ( *K*_D_ ≥ 2.0 × 10 ^3^ nM). C4 did, however, exhibited a comparable affinity for A1 ( *K*_D_ = 139 ± 65 nM), compared with C3 ( *K*_D_ = 252 ± 130 nM) ( Table 1 ). The A2 domain had little binding capacity in ELISA or no binding capacity for either C3 or C4 using SPR.

**Fig. 5:**
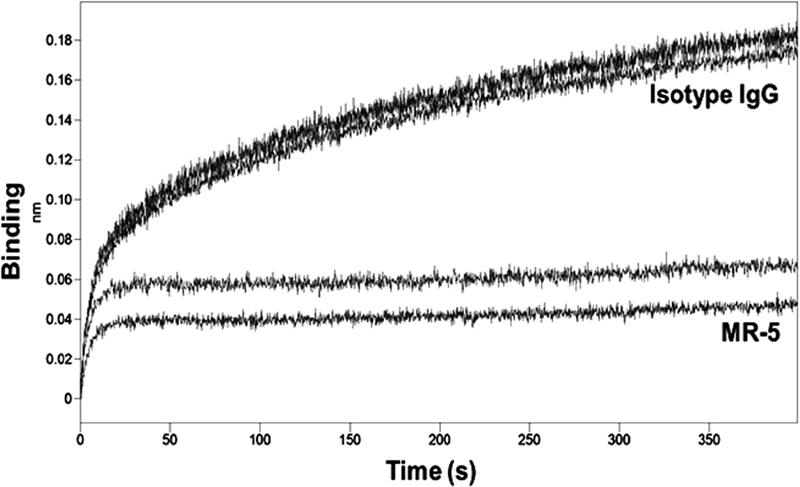
Binding of VWF/ULVWF (ULVWF) to C3 protein is blocked by anti-A3 domain antibody. Biosensors coated with C3 were dipped onto wells containing a solution of ULVWF mixed with mouse IgG (25 μg/mL) or anti-VWF A3 domain antibody, MR-5 (25 μg/mL). The MR-5 antibody inhibited ∼70% of the ULVWF–C3 binding. The graph shows two separated experiments.

The capacity of the antibody MR-5 (monovalent Fab fragment) to block the binding of soluble VWF/ULVWF multimers (labeled “ULVWF”) to C3 was assessed by utilizing BLI technology. Fig. 5 demonstrates that MR-5 was effective in blocking the interaction between VWF/ULVWF in solution and C3 protein bound to Streptavidin biosensors. Thus, the data are compatible, even using different techniques (ELISA methods for Figs. 2 and 3 and an advanced ELISA-like method [Octet Red 384] for Fig. 5 ; surface plasmon resonance for Figs. 4 and Table 1 ).

## Discussion

The precise event initiating AP activation has remained undefined for many years. During this time, it was demonstrated 14 15 16 17 18 that limited activation of the AP could begin by direct hydrolysis of an intramolecular bond in C3. This process converted C3 into a hydrated form [C3(H _2_ O)] that is capable of assuming a conformation that can cleave/activate C3, releasing a 9-kDa fragment (C3a) to form C3b. Amplification of additional C3b generation from C3 requires the binding of C3b [or C3(H _2_ O)] to an “activating cell [surface] macromolecule.” 14 In previous studies, we demonstrated that EC-secreted/anchored ULVWF strings were capable of binding C3 (either in hydrated form or as C3b), and initiating the assembly of the AP. In the current study, our ELISA and surface plasmon resonance experiments demonstrated that C3, C3b, and, to a lesser extent, C4 bind to the recombinant A1A2A3 triple domain (a portion of the VWF monomer); 8 9 C3 and C3b were also capable of binding to the recombinant A3 domain—but not to the A1 or A2 domains.

The binding of C3 to VWF may depend on the conformation of the different proteins. Soluble human VWF/ULVWF multimers, recombinant human smaller VWF multimers, and recombinant human VWF dimers were used initially in pilot experiments on VWF binding to C3 or C4 (results for smaller VWF multimers and dimers are not shown). There was some binding of C3, and to a lesser extent, C4, to the smaller VWF multimers and dimers. We previously reported 4 C3 attachment (and AP activation) on ULVWF multimeric strings that have been secreted by—and *anchored t* o—the surfaces of human ECs. We do not have any data to support the activation of the AP on soluble VWF forms that are not anchored to ECs. In the experiments in this study, the differences in conformation between (glycosylated) VWF A1A2A3 and (nonglycosylated) VWF A3 and between experimental conditions (static vs. flow) probably determine protein–protein affinity. Importantly, the C3 binding to A3 domain in full-length VWF was validated employing a well-characterized monovalent (Fab fragment) antibody against the A3 domain of human VWF, MR-5, 7 that blocked the binding of ULVWF multimers to captured C3 protein.

Here, we show that the A3 domain of VWF contains a major binding site for C3 protein. This result is consistent with a recent study that reported the binding of C3b protein to immobilized recombinant A3 domain protein. 19 With the exception of collagen types I and III 20 21 22 and thrombospondin, 23 24 no other ligands have been described for the A3 domain. This domain does not require a conformational change to enable it to bind to exposed collagen in the subendothelium. That is, the A3 domain in ULVWF strings is already in the conformation that allows collagen binding. However, the A3 domain may exist in high- and low-affinity conformations available for binding to C3 or C3b, as described for the VWF-A-like domain in factor B (FB). 25 26 In fact, the binding site of C3b has been identified in the FB VWF-A-like domain, which has a 15.3% sequence identity with VWF-A3 domain. 27 In previous experiments demonstrating C3 binding to HUVEC-anchored ULVWF strings, 4 the concentration of C3 released into the HUVEC supernatant under the experimental conditions used (1 mL PBS, 15-minute incubation) was below about 5 pM. 28 29 The *K*_D_ of C3 for ULVWF strings would be, therefore, of the order of pM. Our surface plasmon resonance data demonstrate that the *K*_D_ of C3 to A3 or VWF A1A2A3 is approximately 100 to 600 nM. The differences in *K*_D_ suggest that the isolated A3 domain, as well as the A3 domain as a component of A1A2A3, assume a different conformation than the conformation exposed in EC-anchored ULVWF multimeric strings.

The results obtained from using our recombinant A2 domain protein contrast with those recently reported by Bettoni et al. 19 Their study demonstrated the binding of soluble C3b to the three A domains of VWF, and indicated that the main binding site for C3b protein is located in the A2 domain of VWF. In our study, the C3 protein also bound the three A domains, but it had the lowest or no binding activity for the recombinant A2 domain. A possible explanation for this discrepancy is that our A2 is 20 amino acid residues longer than the protein used by Bettoni et al. 19 The additional amino acid residues may alter the structural conformation adopted by the recombinant A2 domain 30 when tested under different conditions (e.g., static [ELISA] vs. flow [SPR]). Another possible explanation for the discrepancy is that we utilized C3 protein, whereas Bettoni et al used C3b protein in their binding analyses. On the other hand, our isolated A2 domain protein had a weak binding activity for C3 in solution (shown in Fig. 3 ), while, in sharp contrast, a protein–protein interaction between the A2 domain and C3 protein was not detected by using SPR. These outcomes suggest that the covalent coupling of either A2 or C3 protein to the chip impairs the recognition sites for the potential A2–C3 binding. Further studies may clarify the results.

Both hydrated C3 (designated “C3” in this study) and C3b may bind to surfaces, including ULVWF-secreted/EC-anchored strings covalently. These covalent bonds may be via an exposed thioester in C3 or C3b to hydroxyl-containing amino acids (threonine, serine, and tyrosine) onto an activating surface 14 (including anchored ULVWF strings). C3 or C3b molecules on anchored ULVWF strings then bind FB to produce C3B or C3bB. 17 18 FB in the C3B or C3bB complex is cleaved to active Bb by factor D, generating C3Bb or C3bBb (the active AP C3 convertases).

Attempts to design drugs targeting the exposed thioester in C3b as a means to inhibit the complement pathways have been explored by Sahu et al, 31 and further studied by Sahu and Pangburn. 32 The cleavage by hydrated C3 of C3 into C3b can be accelerated by the interaction with a surface. 33 ULVWF strings that are secreted by, and anchored to, stimulated human ECs provide important surfaces that promote C3 and AP activation. 34

In our previous experiments using HUVEC-anchored ULVWF multimeric strings, C4 did not bind to the strings. This indicated that neither the classical nor lectin complement pathways were activated by this mechanism. 4 In addition, a recent study also showed that the C4 protein did not bind to individual A1, A2, or A3 domain proteins. 19 In contrast, in the current study we found that C4 had weak binding for the (glycosylated) VWF A1A2A3. C4 did, however, bind to the individual, isolated (nonglycosylated) A1 domain with high affinity (but not to individual A2 or A3 domains). In EC-anchored ULVWF strings, the A1 domain is glycosylated (as in our recombinant VWF A1A2A3). It is likely that glycosylation in A1 domain weakens the binding of C4 to human EC-secreted and EC-anchored ULVWF multimeric strings. By analogy, the glycosylated single A1 domain binds to its major ligand, the platelet receptor glycoprotein (GP) Ib, with a binding affinity that is less than the affinity for nonglycosylated A1 domain. 35 On the other hand, the natural interaction between the A2 domain and A1 domain inhibits the binding of A1 domain to GPIb in the context of VWF A1A2A3 triple domain protein. 8 36 It is well established that immobilization of recombinant VWF A1A2A3 protein disrupts the A2–A1 interaction, exposing the cryptic site for GPIb. 8 37 Thus, it is also probable that this domain–domain interaction blocks the access of C4 for its site within the A1 domain.

In summary, this study reports the differential binding of C3, C3b, and C4 to recombinant glycosylated VWF A1A2A3, and demonstrates that C3 possesses a greater binding affinity for the VWF A3 domain in comparison to C4. Similarly, glycosylated VWF A1A2A3 exhibited a much higher binding affinity for C3 than for C4. These results, together with the capacity of the anti-A3 domain monovalent antibody to block the binding of ULVWF multimers to C3 protein, indicate that the A3 domain in the A1A2A3 complex of ULVWF multimers serves as the docking site where C3 binds and initiates the assembly and activation of the AP. Our experiments suggest that inhibition of the initiation of the AP in inflammatory conditions might be impaired by a compound that blocks the binding of C3 to A3 domains in ULVWF multimeric strings that have been secreted by, and anchored to, human ECs.
